# Digital Health–Enabled Community-Centered Care: Scalable Model to Empower Future Community Health Workers Using Human-in-the-Loop Artificial Intelligence

**DOI:** 10.2196/29535

**Published:** 2022-04-06

**Authors:** Sarah M Rodrigues, Anil Kanduri, Adeline Nyamathi, Nikil Dutt, Pramod Khargonekar, Amir M Rahmani

**Affiliations:** 1 Sue & Bill Gross School of Nursing University of California Irvine, CA United States; 2 Department of Computing University of Turku Turku Finland; 3 Department of Computer Science University of California Irvine, CA United States; 4 Department of Electrical Engineering and Computer Science University of California Irvine, CA United States

**Keywords:** digital health, community-centered care, community health worker, artificial intelligence, AI, AI-enabled health delivery, eHealth, individualized delivery, interventions, collaborative health, community health, social care, digital empowerment, mobile phone

## Abstract

Digital health–enabled community-centered care (D-CCC) represents a pioneering vision for the future of community-centered care. D-CCC aims to support and amplify the digital footprint of community health workers through a novel artificial intelligence–enabled closed-loop digital health platform designed for, and with, community health workers. By focusing digitalization at the level of the community health worker, D-CCC enables more timely, supported, and individualized community health worker–delivered interventions. D-CCC has the potential to move community-centered care into an expanded, digitally interconnected, and collaborative community-centered health and social care ecosystem of the future, grounded within a robust and digitally empowered community health workforce.

## Introduction

### Background

Recent global health trends are necessitating a shift away from a patient-centered medical care system to an upstream health promotion approach that meets the health and social needs of individuals in the communities where they live [1]. A community-centered health and social care ecosystem, supported by a robust community health worker (CHW) workforce, aligns with this needed paradigm shift [1]. Throughout the United States, states are looking to build their CHW workforces and launch CHW initiatives as an alternative assistance to registered health care professionals to extend the reach of home-delivered services [2,3]. Recent data from the California Employment Development Department, for example, project that an additional 200,000 CHWs will be needed by 2024 to maintain current levels of coverage for home care services [4]. As the COVID-19 pandemic continues to amplify health and social support needs globally, a *rapid expansion of the* CHW *footprint is critically needed* to prevent unmet needs from escalating into expensive medical crises, particularly in vulnerable communities [5].

**Figure 1:**
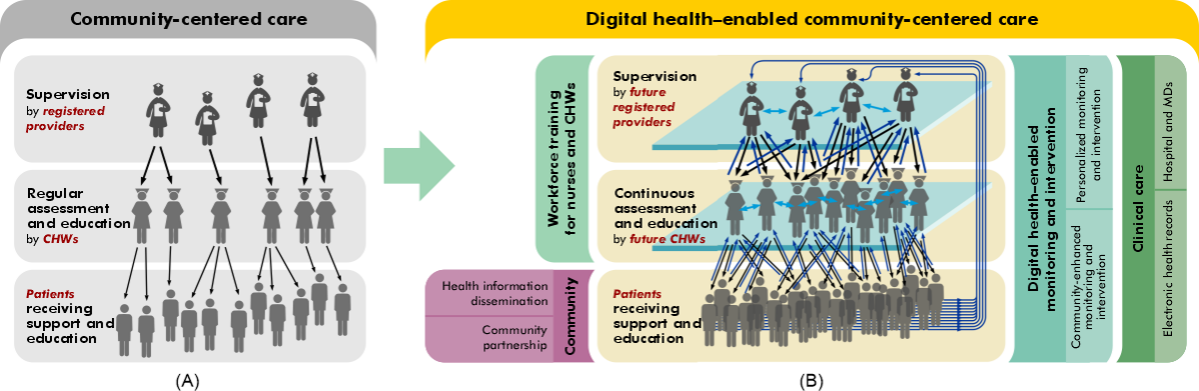
(A) The current community-centered care model. (B) The proposed digital health–enabled community-centered care model. CHW: community health worker; MD: medical doctor.

However, scaling the current community-centered care (CCC) model (Figure 1A) in a cost-effective manner while preserving the highly contextual and individualized care delivered by CHWs remains challenging. Increasing use of digital technologies within the CCC model demonstrate potential to expand the CHW footprint; however, existing digital technologies lack integration. In this paper, we present digital health–enabled CCC (D-CCC) as an improved model to move CCC into the future. Through integrating future digital health technologies, the future CHW workforce, and the future health and social care needs of communities, D-CCC aims to connect the most vulnerable individuals within communities to needed health and social services.

### CHWs as Vital Bridges

CHWs are lay members of the community whose in-depth understanding of community culture and language uniquely positions them to provide culturally appropriate health-related services and support to the community [6]. Through shared lived experience and deep familiarity with social networks and community resources, CHWs serve as vital bridges between vulnerable communities and health systems, connecting community members to critical information, resources, and services [6,7].

CHWs work in a variety of settings—from nonprofit community-based organizations to government agencies and health care systems [7,8]—and range from formal, salaried employees to informal, volunteer-based community educators [9,10]. Just as the activities and roles of CHWs are tailored to the unique health needs of the communities they serve [11], CHWs operate under a diverse set of titles, including but not limited to *promotoras*, community lay workers, outreach educators, peer support workers, home visitors, and community health volunteers [12]. In this paper we use *CHW* as an umbrella term intended to broadly capture the rich diversity of roles and titles held by this vital frontline workforce [6].

As trusted members of the community, CHWs are critically positioned to support vulnerable patient populations [13] and serve as agents of change by helping to reduce health disparities in underserved communities [14]. Documented positive impacts of CHWs include health promotion, improved patient engagement, support with adherence to treatment, improved referrals and access to care, financial return on investment, and improved quality of care and health outcomes [7]. CHW-delivered sociobehavioral interventions have demonstrated efficacy in improving health outcomes in chronic disease and noncommunicable disease (NCD) care and management [15], including cancer [16], diabetes [17-23], asthma [24,25], cardiovascular disease [26], multiple medical comorbidities [27], and mental health [28,29]. CHW-delivered interventions have demonstrated efficacy in reducing hospitalization and rehospitalization rates [27,30-32], and CHWs have proven to be powerful drivers of decreased health care costs, particularly among patients with high starting health care costs and underserved and minority populations [15,17,33,34]. The efficacy of CHWs lies in their close connection to the community, ability to influence client behaviors, and effective interaction with the larger health care team [34-36].

Recent global health trends (including the growing burden of chronic diseases and NCDs), a focus on social determinants of health and health equity, and lessons learned from the COVID-19 pandemic are necessitating a shift away from a patient-centered medical care system toward an upstream health promotion approach that meets the health and social needs of individuals in the communities where they live [1]. In place of the traditionally siloed existence of medicine, public health, and mental health [37], a community-centered health and social care ecosystem, *supported by a robust* CHW *workforce*, will be critical to realizing this shift [1] and preventing unmet needs from escalating into expensive medical crises [5].

## The Current Use of Digital Technologies in CCC

### Overview

Most CHWs operate within a facility-based CCC model (Figure 1A) [38]. Under the CCC model, a relatively smaller number of facility-based supervisors (most often registered health care professionals such as public health nurses, midwives, and community health officers) [38] supervise a relatively larger number of CHWs who, in turn, provide culturally tailored, language-appropriate, and individualized care to a yet larger number of clients. Although this *pyramidal* care model has demonstrated improved health outcomes and cost savings [27,32], scaling this model in a cost-effective manner while preserving the highly contextual and individually tailored care provided by CHWs remains challenging. Facility-based supervisors who themselves typically shoulder a heavy workload may additionally lack the time needed to provide supportive supervision to CHWs [38], resulting in lower quality of supervision [39] and strained CHW-supervisor relationships [40]. However, the recent incorporation of digital health technologies into the existing CCC model is demonstrating potential for expanding the CHW footprint and improving CHW support and supervision. Existing digital technologies are discussed in the following section.

### Current Digital Technologies Used in CCC

The use of digital technologies has recently been increasing among CCC organizations and is demonstrating potential to enhance CHWs’ reach and diffusion of health information within communities [6,41,42]. In their scoping review of the use of mobile health (mHealth) technologies and interventions among CHWs globally, Early et al [6] highlighted some key benefits and challenges. Benefits included promotion of health equity; reduced time to diagnosis; extension of health information and services to diverse areas; improved adherence to treatment plans; increased self-efficacy of patients and CHWs; and improved attitudes of, and toward, CHWs and their role [6]. Challenges included a lack of evaluation of mHealth outcomes; development of mHealth tools and apps without cultural relevance; lack of access to, and knowledge of, mobile technologies within communities; need for effective training for CHWs to adopt mHealth tools; and need for improved communication among health care teams working with CHWs [6]. Similarly, in their narrative review of the literature, Mishra et al [11] identified key benefits and challenges to incorporating digitalization into CHW practice. Benefits included improved access and quality of services, increased efficiency in training and personnel management, and leveraging of data generated across grassroots platforms to further research and evaluation [11]. Challenges included funding for CHW programs; digital health literacy of CHWs; and systemic challenges related to motivating CHWs, including adequate CHW supervision [11].

Digital platforms currently used by CHWs within CCC-based organizations include mobile-based networking devices, web applications, videoconference, and mobile apps [11]. CHWs may use mobile phones, tablets, and other digital devices [43] in a task-specific manner (eg, digital blood pressure [BP] monitoring devices, glucometers, and spirometers) [11,44]. Digital alerts, reminders, notifications, checklists, and decision support tools may be used to facilitate compliance with protocols and improve the quality of CHW-delivered care [11,45]. In addition, although continuous monitoring of physiological parameters (eg, heart rate) has typically been available only in clinical settings because of the need for special equipment and medical expertise, the development of relatively low-cost, nonintrusive sensors present increased opportunities to enable ubiquitous home monitoring of clients’ health and well-being. Current remote sensors distributed and managed by CHWs enable remote monitoring of electrocardiogram (ECG; eg, KardiaMobile [AliveCor]), BP (eg, Evolv [Omron]), blood glucose level, and physical activity [11]. Digital platforms are also being used to facilitate and augment CHW training and supervision [11,46,47] and to provide electronic decision support [11]. Digital platforms that support CHW-CHW communication and collaboration (eg, informal groups and learning networks [48-52]) may provide positive psychological benefits to care workers [50] as well as enable CHWs to exchange information and pose questions to peers [11,53]. Digitalization also presents increasing opportunities for data collection and analysis [11], allowing for critically needed outcome evaluation of CHW-delivered interventions and increasing opportunities for key CHW-focused policy advocacy. However, large-scale data acquisition, preprocessing, and validation for intelligent decision-making remain key challenges with existing methods. Limitations with current approaches are discussed in the following section.

### Limitations of the Current Approaches

Although current digital technologies are demonstrating the potential to strengthen CHW capacity and quality of care [54-56], existing digital technologies targeted for use by CHWs lack integration. The current lack of ubiquitous, closed-loop monitoring and intervention within the CCC model restricts technologies used by CHWs to a passive, episodic, and reactive approach to client monitoring, education, and supportive care. CHWs engaged in NCD management and monitoring, for example, may typically provide infrequent (eg, monthly) home visits and rely on their own episodic observations and client-reported symptoms to inform care, direct education, or make referral decisions. However, direct CHW observation and client reporting of symptoms reveal only episodic snapshots of overall client physical and mental health and lifestyle. Under the CCC model, CHWs rely upon accurate reporting of health symptoms by the client or client caregivers (typically family members) [57] and must also rapidly synthesize their own observations with this information to decide an appropriate course of action (eg, educate, refer, recheck, or call for immediate medical assistance). This places a large burden of responsibility on the CHW, who must synthesize a large amount of information in a short span of time, as well as on the client or caregiver, who must accurately and honestly appraise and report their experiences to the CHW [57]. An integrated, intelligent, and automated closed-loop platform may improve upon this current model of care while maximally amplifying and expanding the CHW footprint within communities.

## A New Model: D-CCC

### Overview

We propose D-CCC as an improved model to move CCC into the future. Design conceptualization for the D-CCC model reflects a multidisciplinary collaboration among nursing, computer science, engineering, and human-computer interaction experts composing the D-CCC team. D-CCC constitutes a novel integrated, intelligent, and automated closed-loop technology platform designed for, and with, CHWs. By targeting the human-technology partnership at the level of the CHW, D-CCC aims to amplify human connection and collaboration while maximally expanding the CHW footprint within communities. By including development of personal models unique to each client, holistically represented within a high-dimensional cover of multiple knowledge layers, D-CCC additionally allows for an increased level of contextual and individualized care delivered by CHWs.

### The D-CCC Model

The D-CCC model transforms manual and restricted aspects of CHW work into a scalable, digital, and intelligently automated space. By expanding CHW-client communication and CHW collaboration, supervision, and support, D-CCC aims to increase the quality of services delivered, in terms of personalization, cultural appropriateness, and timeliness. Through smart supervision, D-CCC may enable CCC organizations to employ and supervise a larger group of CHWs with the same number of CHW supervisors. A central aim of the D-CCC model is to expand the CHW footprint by enabling CHWs to serve a larger volume of clients or to provide more services to each client while critically striving to avoid increasing undue burden on this vital frontline workforce. Through automation of manual tasks; improved communication and connections between CHWs and clients, among CHWs, and between CHWs and supervisors; and increased modalities for ongoing education and training, D-CCC also aims to empower CHWs and improve worker experience.

Figure 1A illustrates the current CCC model. This model lacks integrated digitalization among *tiers* of care delivery and is additionally limited by unilateral communication and poor scalability. In comparison, Figure 1B illustrates the D-CCC model, which addresses these limitations and amplifies the CHW footprint by increasing CHW-client, CHW-CHW, and CHW-supervisor communication and collaboration. D-CCC constitutes a human-technology partnership integrating CHWs, supervisors, and clients through a scalable digital medium and aims to improve the efficiency and quality of care delivery. Through an intelligent and scalable artificial intelligence (AI) loop, D-CCC brings critical stakeholders under a unified communication, collaboration, education and training, and care delivery model.

### D-CCC’s AI-Enabled Closed-loop Health Platform

As CHWs occupy a variety of roles within communities and as CHW-delivered interventions and target outcomes vary according to the client populations served, a *one-size-fits-all* platform design will not be successful in empowering this diverse frontline workforce. D-CCC design must therefore be modular and allow for ongoing adaptability and flexibility. As cultural and contextual needs will vary according to the populations served, D-CCC design must also allow for portability across diverse communities, including translation and transferability to different languages spoken in local communities.

The D-CCC platform is designed to meet the diverse needs of different stakeholders, including clients, CHWs, and registered providers (RPs), and to handle diverse aspects such as communication among stakeholders, data collection and analysis, training and education, and autonomous and intelligent decision-making.

The D-CCC platform design is built on the following modules:

1. Multichannel stakeholders module: This module comprises the stakeholders who are interacting through different communication channels (including client-CHW, CHW-CHW, and CHW-supervisor) and enables the smart communication aspects of D-CCC.
2. Monitoring module: This module enables continuous monitoring and updating of physiological and contextual information collected both subjectively and objectively.
3. Health estimation module: This module enables ongoing analysis of the data collected from clients (through the monitor module, CHWs, and medical history) to determine client health status in real time.
4. Knowledge base module: This module manages the storage, access, and retrieval of built knowledge based upon data collected from clients, CHWs, and supervisors to enable personalized model building.
5. Personal models module: This module builds cognitive learning models from client physiological and contextual data to provide autonomous intelligent decisions for intervention recommendations individually tailored for each client.
6. Smart recommendation module: This module autonomously predicts appropriate health care and lifestyle recommendations for clients leveraging their personalized models. Recommendations may be delivered to CHWs or directly to clients, according to context and risk level.
7. Smart supervision module: This module supports RPs in supervising CHWs in specific areas determined through automating repetitive tasks and reflecting on personal models and the knowledge base.
8. Smart assistance module: This module improves quality of health care services by supporting CHWs making interventional, educational, or procedural decisions in the field.
9. Smart training module: This module provides training for CHWs to learn new digital technologies and enables web-based training modules to augment traditional, in-person methods of CHW training and education.

**Figure 2:**
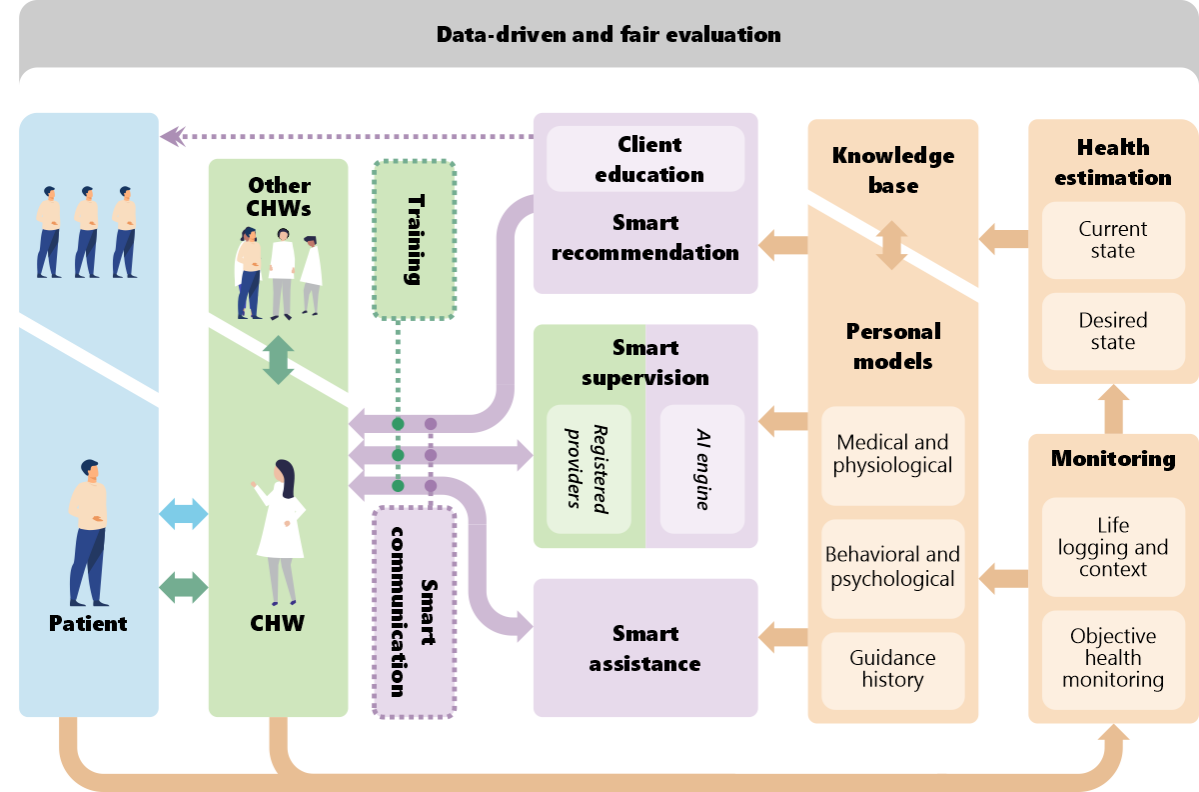
The digital health–enabled community-centered care model’s artificial intelligence (AI)-enabled cybernetic platform. CHW: community health worker.

As shown in Figure 2, D-CCC’s AI-enabled platform critically integrates these modules to create a holistic and data-driven approach to connect clients, CHWs, and supervisors in a continuous loop of measurement, estimation, guidance, and influence. Each module is further described in the following sections.

### Multichannel Stakeholders Module

The D-CCC model critically enables multi-way communication among key stakeholders involved in CCC delivery networks through digital communication channels. Figure 2 shows the different combinations of multi-way communication channels among clients, CHWs, and supervisors, which can be further classified into the following communication channels:

1. Client-CHW channel: This is the point of communication between clients and CHWs. As a baseline, each client is assigned a CHW who is responsible for providing community health-related services. Traditionally, the client-CHW interaction is scheduled over fixed time windows or on demand, subject to the client’s needs. D-CCC transforms this into a continuous interaction, and the digital communication medium may be made available to all clients served.
2. CHW-supervisor channel: This is the point of communication between CHWs and supervisors. As a baseline, each CHW has a supervisor assigned for supervision and feedback. As service delivery demands change, CHW supervision can be extended to include additional supervisors (eg, RPs) with expertise in different areas of care. D-CCC transforms CHW supervision to an on-demand, real-time interaction through the digital medium.
3. CHW-CHW channel: This is the point of communication among CHWs. Communication and collaboration among CHWs promote knowledge transfer and exchange of information, which may be adapted to different community-specific challenges as needed. D-CCC transforms CHW-CHW communication into an on-demand, real-time interaction through the digital medium.

### Monitoring Module

This module is responsible for continuous monitoring of client physiological signs, contextual information, lifelogs, surveys, and ecological momentary assessments. State-of-the-art wearable (eg, smart rings, watches, and patches), portable (eg, smart ECG sensors, stethoscopes, and BP monitors) and stationary sensors (eg, smart beds and fall detection cameras) as well as mHealth (smartphone-based) solutions enable ubiquitous monitoring. D-CCC transforms the current landscape of disparately monitored parameters by providing a central collection point from which subsequent data integration and synthesis enable provision of smart recommendations and assistance to key stakeholders.

### Health Estimation Module

This module is responsible for determining the health status of the client. High-dimensional and holistic information collected and processed by this module identifies the client health state in real time. Each client is unique and presents multiple possibilities of health states. A key functionality of this module is to process different modalities to properly estimate health variables in different dimensions. For each client, this module holds a health status that is deemed to be safe, termed the client’s *desired state*. This module continuously estimates the current state (ie, client’s current health status) and compares the current state with the desired state to estimate the overall health safety of the client. Client health status as determined by this module assists in building personal models and guides decision-making by recommendation engines.

### Knowledge Base Module

**Figure 3:**
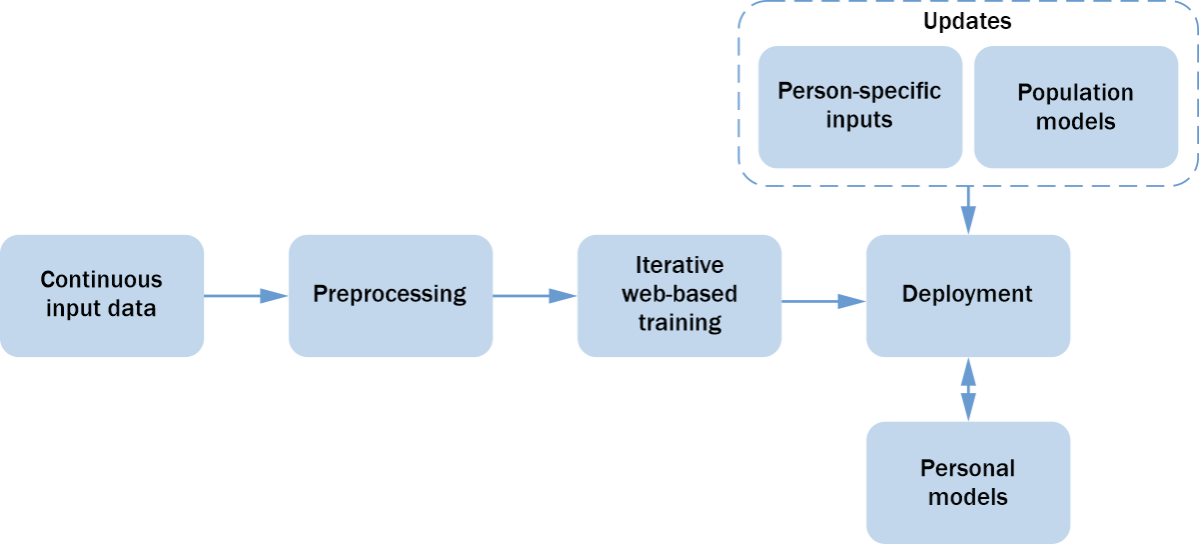
Deployment pipeline for personalized models.

This module consists of CCC-related facts, information, and skills acquired through past experience of different stakeholders (CHWs, supervisors, and clients), as well as theoretical or practical knowledge available in the CCC field. This module continuously learns and updates its knowledge based on new inputs extracted from the data generated by the entire pool of clients, CHWs, and supervisors. The knowledge base formulates knowledge graphs that represent different meaningful connections between cause-action pairs to develop a general consensus. For example, specific readings in physiological data can be connected to specific health criticality. The knowledge graphs will form the basis for developing population models that guide personal model development. Thus, the knowledge base implicitly enables reuse of key insights from a specific individual to develop comprehensive personal models for other individuals. Figure 3 illustrates the pipeline of deploying a generic learning analytics model, which can be updated with person-specific information to generate personalized models. Each client’s data include physiological parameters monitored continuously and contextual information associated with each instance of lifelogging, as well as intervention procedures performed in the past and health estimation logs. Each CHW’s data include the list of assigned clients, log of interventions performed per client, and effectiveness of these interventions, as well as supervision and training provided by supervisors. This module guides the building of learning analytics and cognitive models in the subsequent modules.

### Personal Models Module

This module builds analytical models for intelligent decision-making tailored to a specific individual based on a *predictive, preventive, personalized, and participatory* (P4) [58] approach to health care. The P4 approach holds that each individual constitutes a biological system that responds differently to different inputs [58]. To build an approach that makes P4 a persistent action to guide CHWs and clients, it is important to estimate and build a personal model of each client. In many areas relevant to personalization, a model is built by collecting data in the context of the application. Examples of this approach include Google, Facebook, Amazon, and Netflix, which generate personalized recommendations using models built for each individual when they interact with different applications on the platforms. The D-CCC personal models module relies on the physiological parameters, context, and health status data of each client collected by the monitoring module and the health estimation module. A variety of methods, such as machine learning, can be used for data analysis and cognitive modeling for autonomous decision-making. Figure 3 shows the deployment pipeline of individual learning models and person-specific input data to generate personal models. In the preprocessing phase, input data from different modalities are filtered to remove noisy components and motion artifacts. We use collaborative filtering to handle missing values from the data set of a specific modality by complementing the missing data with insightful information from other modalities. In the iterative web-based training phase, each modality of input data sets is used to train a suitable learning model that fits the corresponding modality. The trained models that are deployed in the initial phase are continuously updated with the iterative web-based training. From the knowledge base, we also create population models that contain heuristics to formulate rules for updating trained models with generalized person-specific inputs. As we collect person-specific data, the trained models are updated with these person-specific inputs to generate the personal models. The personal models are updated with new person-specific inputs every 6 months. As shown in Figure 2, we divide personalized modeling into three submodules, as described in the following paragraphs:

1. Medical and physiological profile: This submodule builds a personalized medical and physiological model for each client from the physiological data and health status estimate. This creates a baseline for each client’s health status and provides an intuition on determining the relative health safety under the current conditions. For example, although two clients, A and B, may have a history of the same condition, the specific health status of client A can be perceived as more alarming than that of client B based upon their personalized medical and physiological profiles.
2. Behavioral and psychological profile: This submodule builds a personalized behavioral and psychological model for each client from contextual data. This creates an insightful perspective on each client’s health status and their response to interventions under specific circumstances. For example, a situation where two clients, A and B, have the same health status but different contexts would call for different intervention responses (eg, education or referral procedures). This allows CHWs to choose among actions that are customized to both the client’s physiological status and their context.
3. Guidance history: This submodule logs the past health conditions of a client along with their contexts, the responsive intervention procedures imposed under each context, and the response of the client to each intervention use case. This allows for development of a baseline understanding for CHWs to decide on appropriate action in the current scenario. Given the history of interaction and guidance provided by CHWs in the past under similar situations, decisions on an improvised intervention or on reusing a previously successful intervention can be made.

We use multimodal data fusion strategy to combine the results from each of the aforementioned modalities of personal models. Data fusion improves the prediction accuracy of the personal models, particularly in instances where input data are missing from specific modalities.

### Smart Recommendation Module

This module delivers autonomous health recommendations to clients and/or CHWs based on risk level and recommendation type. D-CCC combines each client’s physiological and contextual data from the knowledge base module and personal models module to build smart recommendation systems. Smart recommendation is a proactive strategy to promote client self-management and improve quality of life, particularly in less-acute scenarios (eg, recommendations related to diet, physical activity, sleep, stress, and medication reminders). Smart recommendations sent to clients target everyday health maintenance and education regarding healthy life choices. This module infers to the cognitive and analytical models from the personal models module to make autonomous recommendations. The autonomous recommender primarily aims to bridge the gap between the increasing number of clients in need and the relatively lower number of CHWs and RPs and is based upon the acuity of client condition. Recommendations are also sent out to the responsible CHW to synchronize the information flow. The decision regarding whether a recommendation is delivered directly to the CHW, directly to the client, or delivered through the CHW to the client depends on the settings and recommendation type. For instance, some client populations (eg, older adults) with less access to, or comfort using, smartphones may prefer to receive recommendations through their CHWs, whereas other client populations (eg, pregnant women) with greater access to, or comfort using, smartphones may prefer to receive health promotion recommendations directly. This module adds a layer of cyber health care service delivery to D-CCC, enabling safe and timely interventions for different clients with varied needs.

### Smart Supervision Module

This module automates workflow between supervisors (eg, RPs) and CHWs to improve supervision efficiency both qualitatively and quantitatively. There is a need for ongoing CHW-supervisor interaction for evaluation, feedback, and support. Under the current CCC model, supervisors manually organize supervisory tasks with different CHWs. D-CCC enables automated supervision support using AI models. Each CHW handling multiple clients in different contexts presents each RP with different supervisory challenges. The personal models module and knowledge base module contain cognitive analytical models about different clients. The smart supervision module integrates these personalized models into an AI engine that serves as the oracle to supervisors. This allows supervisors to hone their attention to specific issues that each individual CHW may have while accessing the collective record of clients specific to that CHW. In addition, enabling a common and more complete understanding of the clients’ contexts may enable interactions between RPs and CHWs to become more qualitative while reducing overhead time. Supervisors may additionally choose to automate trivial repetitive tasks, allowing them to supervise a larger number of CHWs or provide more personal support and mentorship to an existing cadre of CHWs. Multi-way communication among CHWs and supervisors additionally allows for a range of choices to match supervision requirements of CHWs to expertise of the RPs. Effective supervision is an essential element of CHW programs [38]. Through improving CHW support and enabling more holistic CHW-supervisor interaction, D-CCC may enable scalability of CHW supervision strategies, leading to improved productivity, care delivery, and worker satisfaction.

### Smart Assistance Module

This module supports CHWs to deliver continuous high-quality care services in the field, particularly in scenarios where CHWs may have limited access to resources. Applying intervention procedural knowledge for clients with diverse contexts can be challenging for CHWs in the field, and D-CCC supports CHWs to make client-specific decisions using personalized models. Smart assistance is a reactive and on-demand service intended to handle acute scenarios that may enhance decision-making of CHWs in the field when they do not have immediate access to supervisors; one approach includes implementing customized chatbots used by CHWs in real time to provide appropriate decisions regarding client care (eg, education, CHW-delivered intervention, and referral). This may be particularly useful when supervisors are not accessible or when CHWs encounter a situation with which they have limited expertise. Chatbots use natural language processing to parse textual information provided by CHWs to make sense of the client care situation. This module accesses the client’s medical and physiological profile, behavioral and psychological profile, and previous history of CHW assistance provided from the knowledge base. This module identifies the specific client and infers the client’s personalized model to make autonomous decisions regarding CHW-delivered care.

### Smart Training Module

This module enables ongoing training for CHWs to learn new digital technologies and augments traditional methods of CHW training and education for connected web-based and blended learning methods. Key challenges identified in the literature regarding current use of digital technologies include CHW digital health literacy [11] and the need for effective training for CHWs to adopt digital tools [6]. This module integrates ongoing CHW digital health training and provides CHWs with an automated technology *help desk* for ongoing additional assistance as needed. This module additionally augments traditional, in-person CHW training by providing ongoing case-based digital learning modules, which may be assigned by CHW supervisors and completed by CHWs in an independent and self-paced manner.

## Case Study: Smart, Connected, and Coordinated Maternal Care for Underserved Communities

The D-CCC model is currently being piloted with community partner site MOMS Orange County (MOMS OC) [59]. We outline this proof-of-concept case study in the following sections.

### MOMS Orange County

**Figure 4:**
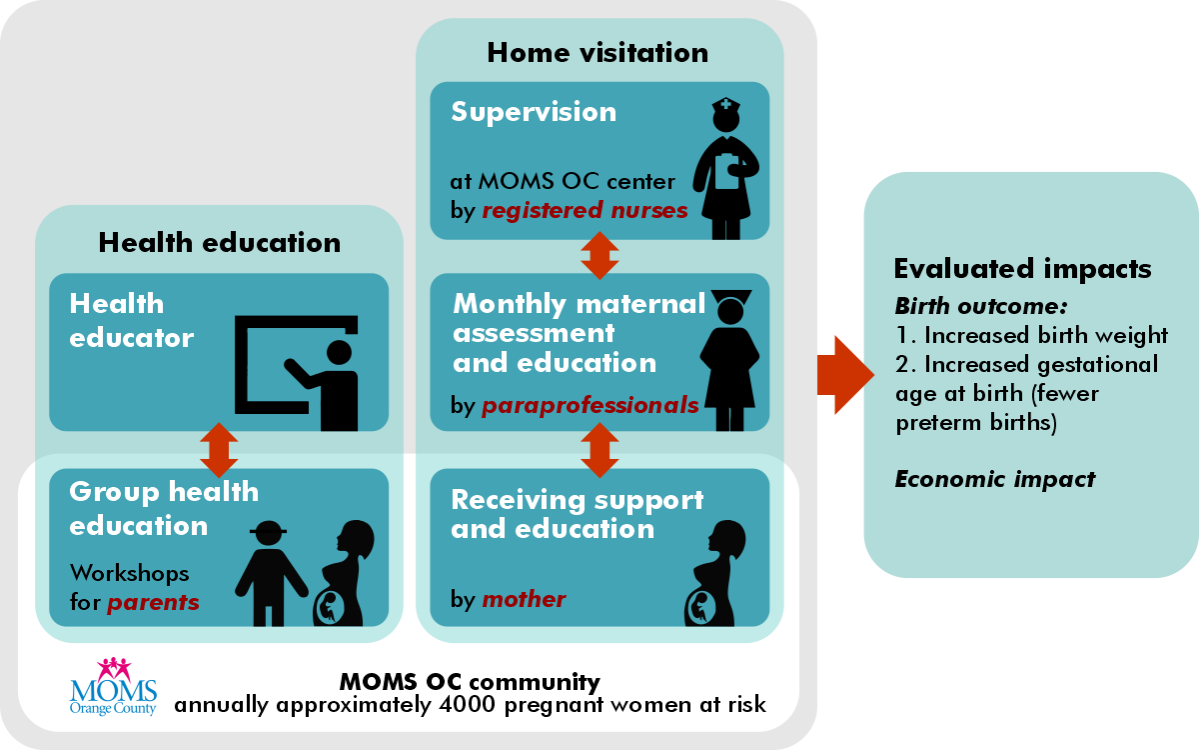
MOMS OC (Orange County) community-centered care model.

Registered nurse (RN)-delivered home visits for at-risk pregnant women became infeasible in the early 1990s in Orange County, California, because of the county’s financial constraints. In response to the county’s maternity care crisis, MOMS OC [59] was founded. MOMS OC is a nonprofit organization implementing a CCC model (Figure 4) for maternal care (MC) delivery. MOMS OC’s CCC model is a CHW-delivered care coordination and home visitation program wherein RNs supervise and train CHWs, who in turn provide culturally and linguistically appropriate services, conduct home visits, and deliver group education to pregnant women considered to be at low to moderate risk and served by MOMS OC.

The MOMS OC CCC model has demonstrated cost efficiency [60] and improved birth outcomes [61]. However, it remains limited by barriers and gaps common to CCC models, which presents opportunities for the incorporation of technology. Barriers and gaps applicable to MOMS OC include the following:

1. *Limitations regarding outreach* using traditional mechanisms (eg, word of mouth, telephone calls, and advertisements), particularly among disadvantaged communities. This presents opportunities for technology-enabled community outreach.
2. *Limitations regarding timely intervention*, including early MC and education. This presents opportunities for new community-driven intervention enablement assisted by digital and social media.
3. *Limitations regarding monitoring* because infrequent CHW home visits (typically conducted monthly) and clients’ self-reported screenings both reveal only a snapshot of each woman’s physical and mental health and lifestyle. This presents opportunities for incorporation of low-cost, nonintrusive wearable devices to enable ubiquitous monitoring.
4. *Limitations regarding communication among MC providers*, with CHWs typically unable to access health data stored as electronic health records and MC providers typically unable to easily access information collected by CHWs. This presents opportunities for networking, smart data mining, and community-enhanced recommendation systems.

### A D-CCC Pilot Community Engagement Model for MC

Identifying opportunities for D-CCC to overcome these gaps and improve the quality of care delivered, we partnered with MOMS OC to launch Smart, Connected, and Coordinated Maternal Care for Underserved Communities (UNITE) [62], a D-CCC pilot community engagement model for MC that is smart (deploying ubiquitous monitoring and lifelogging), connected (bringing together a diverse cast of community members, including clients, families, RPs, and community resources), and coordinated (using technology to proactively reach out to the community and deliver personalized interventions and education for each client).

UNITE deploys technology-enhanced community care coordination and education coupled with a human-in-the-loop monitoring and intervention system to (1) proactively reach out to the community (pregnant women, families, and friends); (2) provide valuable personalized and community-enhanced information for CHWs and RNs to provide tailored support according to individual needs; and (3) combine fine-grained and personalized information together with monitoring and intervention and community outreach and education to promote healthier lifestyles for pregnant women through self-management.

UNITE leverages emerging technologies such as Wearable Internet of Things, community-enhanced learning and recommendation, big data analytics, context recognition, lifelogging, and social media, as well as a multidisciplinary partnership to bring connectivity, integration, and smartness to MOMS OC’s existing CCC model. UNITE also brings ubiquitous monitoring, open information sharing, and community-enhanced personalized intervention and education to MOMS OC’s CCC model. UNITE aims to strengthen connectivity among stakeholders and to improve connection and coordination across providers and agencies focused on pre- and postnatal health.

Through lifelogging, context recognition, and health monitoring, UNITE builds a holistic digital phenotype of participants using multimodal data capture. Smart mining algorithms are designed for cause assessment through personalized models. Maternal self-management is improved and incentivized through personalized community-enhanced recommendation systems and technology-enhanced community care coordination and education. The core components of the UNITE model are ubiquitous monitoring and a recommendation system capable of dynamically supporting a healthy lifestyle of enrolled women during and after pregnancy. UNITE recognizes how community-specific factors pertaining to each client enhance individual monitoring and interventions and enable more personalized recommendations to motivate better self-management. UNITE thus integrates culture- and context-sensitive mechanisms to enhance technology acceptance for improved maternal self-management and enhances connection and coordination with CHWs and other care providers and agencies focused on improving pre- and postnatal maternal health.

As part of the UNITE project, a proof-of-concept version of the AI-enabled closed-loop D-CCC platform has been implemented whereby a Wearable Internet of Things–based health-monitoring flexible and adaptable platform enables seamless capture of sensory data streams (using wearables and smartphones) and self-reported data from participants (using questionnaires and ecological momentary assessments). UNITE’s dashboard allows for interaction between CHWs and the enrolled women, provides visualization and interaction with the backend system, and performs sophisticated analytics and data mining to build a holistic personal model of each participant. Each personal model combines physical and psychosocial data capture to generate feedback and recommendations to the enrolled women, their CHWs, and the CHW supervisors, as well as to other health providers with the goal of promoting healthy pregnancy outcomes.

An exemplar intervention delivered through the UNITE platform’s closed-loop system is a home-based safe exercise program overseen remotely by CHWs [63]. This evidence-based exercise program, designed using the pregnancy and postpartum exercise guidelines provided by the American College of Obstetricians and Gynecologists, includes strength movement, aerobic movement, and mindful breathing. UNITE’s real-time monitoring of intensity and duration of exercise using wearables (eg, by measuring heart rate elevation, maximum oxygen uptake [VO_2max_], respiratory rate, and ratings of perceived exertion) critically enables individualization of the intervention as well as safety monitoring throughout the exercise program.

Completion of the UNITE pilot study will yield critical information regarding feasibility and user (client, CHW, supervisor, and RP) experience as well as effects on health outcomes, including cardiovascular response, sleep, physical activity, self-reported stress, depression, and anxiety.

## Discussion

### D-CCC as an Improved Model to Move CCC Into the Future

As current global health trends necessitate a shift away from a patient-centered medical care system to an upstream health promotion approach that meets the health and social needs of individuals in their communities, a community-centered health and social care ecosystem, supported by a robust and digitally connected CHW workforce [1], is needed to launch CCC into the future. A rapid expansion of the CHW footprint is critically needed to prevent unmet needs from escalating into expensive medical crises, particularly in vulnerable communities [5]. However, scaling the current CCC model in a cost-effective manner while preserving the highly contextual and individually tailored care provided by CHWs remains challenging. Although the recent incorporation of digital technologies into the CCC model demonstrates potential for expanding the CHW footprint, current technologies lack integration. Large-scale data acquisition, preprocessing, and validation for intelligent decision-making remain key challenges.

We propose D-CCC as an improved model to move CCC into the future and present our UNITE pilot study as an initial proof of concept in D-CCC implementation. Of note, UNITE represents *one iteration* of the D-CCC platform, designed to support the specific needs of a community partner organization providing CHW-delivered interventions to pregnant women considered to be at low to moderate risk within Orange County, California. UNITE was designed to leverage technology to facilitate client self-management as well as CHW-delivered support. However, D-CCC may also support CHW-delivered health-related interventions among client populations less able to engage in self-management or with less access to, or comfort with, technology (eg, older adults with dementia). Critically, because CHWs occupy a variety of roles within communities and because CHW-delivered interventions and target outcomes vary widely according to the client populations served, a *one-size-fits-all* platform design will not be successful in empowering the CHW workforce of the future. D-CCC is designed to be scalable and portable across diverse communities, and its modular design purposively allows for flexibility to meet the needs of different CCC organizations, populations served, and care delivery focus.

D-CCC’s design addresses key challenges identified in the literature regarding current use of digital technologies by CHWs, including a lack of evaluation of outcomes; development of digital tools and apps without cultural relevance; lack of access to, or knowledge of, mobile technologies within communities; need for effective training for CHWs to adopt digital tools; inadequate CHW supervision; and the need for improved communication among health care teams working with CHWs [6,11]. Furthermore, D-CCC allows for fair and data-driven evaluation of CHW-delivered interventions, thereby increasing opportunities for key CHW-focused policy advocacy. Data-driven evaluation of CHW-delivered interventions is key to moving community organizations away from fee-for-service or grant-based governmental support models and toward value-driven models for CHW reimbursement.

However, there remain key challenges and limitations to consider. D-CCC design focuses on supporting CHWs operating within a facility-based CCC model of care delivery. Although most CHWs operate under this model [38], exploring how D-CCC might apply to other models of CHW-delivered community care remains a future endeavor. Certain aspects of digitalization (eg, BP monitors, ECG sensors, and blood glucose monitoring) may be more useful for CHWs focused on single or multi-disease management, and ubiquitous monitoring may require clients to use a smartphone and have home internet access. However, D-CCC is designed to be used by CHWs engaged in various community care roles, including use in client populations without access to smartphones or home internet. Through facilitating CHW communication (with clients, supervisors, and other CHWs), collaboration, and knowledge sharing, as well as CHW supervision, training, and decision support, D-CCC is designed to support CHWs engaged in myriad care roles. CHWs working on upstream health determinants such as job loss may also benefit from the D-CCC intelligent and integrated closed-loop digital platform designed to support communication, collaboration, and knowledge sharing of community-based resources.

In addition, the UNITE pilot is presented as a proof-of-concept case study to illustrate D-CCC deployment. However, this pilot is ongoing and limited by a lack of outcome data to present here (eg, regarding feasibility, user experience, or health outcomes). We highlight the larger need for studies conducting cost analyses of CHWs, digital technologies, and the combination of CHWs *and* digital technologies. Without these data, assertions regarding cost savings through incorporation of technologies targeted for use by CHWs remain speculative.

AI design must carefully consider the impact of digitalization on CHW relationships, workload, and workflow [64]. Deployment of AI systems will result in some additional work, most notably upon rollout and initial deployment. Some of this work may be readily apparent (eg, CHWs actually using the AI platform), but less-apparent aspects must also be considered (eg, CHWs introducing, explaining, and justifying the use of new technologies to their communities) [64]. As Okolo et al [64] highlight, AI developers must account for such added visible and invisible work shouldered by CHWs when assessing the benefits and limitations of incorporating AI systems into established CCC workflows. To this end, CHW collaboration and iterative feedback drive D-CCC design to maximize utility and acceptability while minimizing additional worker burden. Our multidisciplinary team also includes human-computer interaction experts to conduct ongoing assessments of CHW relationships and workflows during D-CCC design and implementation.

Finally, D-CCC’s data-driven algorithms, key to building personalized models, also mean an unprecedented scope with regard to sensitive data collection, and the digital health ecosystem presents new ethical challenges and considerations, particularly in vulnerable populations. The D-CCC platform must critically protect data privacy and security through methods such as end-to-end encryption and distributed security schemes (eg, blockchain technology) and proper data governance policies and tools. Any commercial cloud infrastructure services used to store and manage data resources must provide Health Insurance Portability and Accountability Act–compliant services for storage and transmission of protected health information. Data privacy and shared responsibility clauses must additionally guarantee secure data management in compliance with the user and application deployer.

Although new human-technology partnerships have incredible potential to empower future workers and to promote health equity, the impacts of AI on future workers and communities must be considered, including identifying potential for inadvertent creation or exacerbation of health inequities, particularly among vulnerable populations. Consideration of sociocultural aspects throughout D-CCC platform design and iteratively examining how these affect platform acceptability, feasibility, and reliability will be critical to avoid further propagating health inequities [65]. To this end, D-CCC design and implementation must occur iteratively and in close collaboration with all stakeholders, including CHWs, CHW supervisors, and clients.

### Conclusions

The increasing use of digital technologies among CCC organizations is demonstrating the potential to expand the CHW footprint by enhancing CHWs’ reach and diffusion of health information within communities [6,41,42]. However, existing digital technologies incorporated into CHW practice lack integration. We propose D-CCC as an improved model to move CCC into the future. D-CCC constitutes a human-technology partnership integrating CHWs, CHW supervisors, and communities through a scalable digital medium to improve the efficiency and quality of care delivery. By targeting a human-technology partnership at the level of the CHW, D-CCC aims to amplify human connection and collaboration while maximally expanding the CHW footprint within communities. Through integrating future digital health technologies, the future CHW workforce, and the future health and social care needs of communities, D-CCC aims to connect the most vulnerable individuals within communities to needed resources and health and social services.

## Abbreviations

AI: artificial intelligence
BP: blood pressure
CCC: community-centered care
CHW: community health worker
D-CCC: digital health–enabled community-centered care
ECG: electrocardiogram
MC: maternal care
mHealth: mobile health
MOMS OC: MOMS Orange County
NCD: noncommunicable disease
P4: predictive, preventive, personalized, and participatory
RN: registered nurse
RP: registered provider
UNITE: Smart, Connected, and Coordinated Maternal Care for Underserved Communities

## References

[1] Goldfield NI, Crittenden R, Fox D, McDonough J, Nichols L, Lee Rosenthal E (2020). COVID-19 crisis creates opportunities for community-centered population health: community health workers at the center. J Ambul Care Manage.

[2] Salem BE, Klansek E, Morisky DE, Shin SS, Yadav K, Chang AH, Nyamathi AM (2020). Acceptability and feasibility of a nurse-led, community health worker partnered latent tuberculosis medication adherence model for homeless adults. Int J Environ Res Public Health.

[3] (2017). State Community Health Worker Models. National Academy for State Health Policy.

[4] Thomason S, Bernhardt A (2017). California’s homecare crisis: raising wages is key to the solution. UC Berkeley Center for Labor Research and Education.

[5] Waters R (2020). Community workers lend human connection to COVID-19 response. Health Aff.

[6] Early J, Gonzalez C, Gordon-Dseagu V, Robles-Calderon L (2019). Use of mobile health (mHealth) technologies and interventions among community health workers globally: a scoping review. Health Promot Pract.

[7] Lloyd J, Davis R, Moses K (2020). Recognizing and sustaining the value of community health workers and promotores. Center for Health Care Strategies.

[8] (2018). Community health workers in North Carolina: creating an infrastructure for sustainability. NC Department of Health and Human Services.

[9] Zambruni J, Rasanathan K, Hipgrave D, Miller NP, Momanyi M, Pearson L, Rio D, Romedenne M, Singh S, Young M, Peterson S (2017). Community health systems: allowing community health workers to emerge from the shadows. Lancet Glob Health.

[10] Agarwal S, Sripad P, Johnson C, Kirk K, Bellows B, Ana J, Blaser V, Kumar MB, Buchholz K, Casseus A, Chen N, Dini HS, Deussom RH, Jacobstein D, Kintu R, Kureshy N, Meoli L, Otiso L, Pakenham-Walsh N, Zambruni JP, Raghavan M, Schwarz R, Townsend J, Varpilah B, Weiss W, Warren CE (2019). A conceptual framework for measuring community health workforce performance within primary health care systems. Hum Resour Health.

[11] Mishra SR, Lygidakis C, Neupane D, Gyawali B, Uwizihiwe JP, Virani SS, Kallestrup P, Miranda JJ (2019). Combating non-communicable diseases: potentials and challenges for community health workers in a digital age, a narrative review of the literature. Health Policy Plan.

[12] Hodgins S, Kok M, Musoke D, Lewin S, Crigler L, LeBan K, Perry HB (2021). Community health workers at the dawn of a new era: 1. Introduction: tensions confronting large-scale CHW programmes. Health Res Policy Sys.

[13] Brown O, Kangovi S, Wiggins N, Alvarado CS (2020). Supervision strategies and community health worker effectiveness in health care settings. NAM Perspect.

[14] (2014). Role of community health workers. National Institutes of Health.

[15] Kim K, Choi JS, Choi E, Nieman CL, Joo JH, Lin FR, Gitlin LN, Han H (2016). Effects of community-based health worker interventions to improve chronic disease management and care among vulnerable populations: a systematic review. Am J Public Health.

[16] Roland KB, Milliken EL, Rohan EA, DeGroff A, White S, Melillo S, Rorie WE, Signes CC, Young PA (2017). Use of community health workers and patient navigators to improve cancer outcomes among patients served by federally qualified health centers: a systematic literature review. Health Equity.

[17] Brown III HS, Wilson KJ, Pagán JA, Arcari CM, Martinez M, Smith K, Reininger B (2012). Cost-effectiveness analysis of a community health worker intervention for low-income Hispanic adults with diabetes. Prev Chronic Dis.

[18] Carrasquillo O, Lebron C, Alonzo Y, Li H, Chang A, Kenya S (2017). Effect of a community health worker intervention among latinos with poorly controlled type 2 diabetes: the miami healthy heart initiative randomized clinical trial. JAMA Intern Med.

[19] Nelson K, Taylor L, Silverman J, Kiefer M, Hebert P, Lessler D, Krieger J (2017). Randomized controlled trial of a community health worker self-management support intervention among low-income adults with diabetes, Seattle, Washington, 2010-2014. Prev Chronic Dis.

[20] Prezio EA, Cheng D, Balasubramanian BA, Shuval K, Kendzor DE, Culica D (2013). Community Diabetes Education (CoDE) for uninsured Mexican Americans: a randomized controlled trial of a culturally tailored diabetes education and management program led by a community health worker. Diabetes Res Clin Pract.

[21] Vaughan EM, Johnston CA, Cardenas VJ, Moreno JP, Foreyt JP (2017). Integrating CHWs as part of the team leading diabetes group visits: a randomized controlled feasibility study. Diabetes Educ.

[22] Norris SL, Chowdhury FM, Van Le K, Horsley T, Brownstein JN, Zhang X, Jack L, Satterfield DW (2006). Effectiveness of community health workers in the care of persons with diabetes. Diabet Med.

[23] Spencer MS, Rosland A, Kieffer EC, Sinco BR, Valerio M, Palmisano G, Anderson M, Guzman JR, Heisler M (2011). Effectiveness of a community health worker intervention among African American and Latino adults with type 2 diabetes: a randomized controlled trial. Am J Public Health.

[24] Postma J, Karr C, Kieckhefer G (2009). Community health workers and environmental interventions for children with asthma: a systematic review. J Asthma.

[25] Raphael JL, Rueda A, Lion KC, Giordano TP (2013). The role of lay health workers in pediatric chronic disease: a systematic review. Acad Pediatr.

[26] Allen JK, Himmelfarb CR, Szanton SL, Bone L, Hill MN, Levine DM (2011). COACH trial: a randomized controlled trial of nurse practitioner/community health worker cardiovascular disease risk reduction in urban community health centers: rationale and design. Contemp Clin Trials.

[27] Kangovi S, Mitra N, Grande D, Huo H, Smith RA, Long JA (2017). Community health worker support for disadvantaged patients with multiple chronic diseases: a randomized clinical trial. Am J Public Health.

[28] Barnett ML, Gonzalez A, Miranda J, Chavira DA, Lau AS (2018). Mobilizing community health workers to address mental health disparities for underserved populations: a systematic review. Adm Policy Ment Health.

[29] Spencer MS, Hawkins J, Espitia NR, Sinco B, Jennings T, Lewis C, Palmisano G, Kieffer E (2013). Influence of a community health worker intervention on mental health outcomes among low-income Latino and African American adults with type 2 diabetes. Race Soc Probl.

[30] Kangovi S, Mitra N, Grande D, White ML, McCollum S, Sellman J, Shannon RP, Long JA (2014). Patient-centered community health worker intervention to improve posthospital outcomes: a randomized clinical trial. JAMA Intern Med.

[31] Jack HE, Arabadjis SD, Sun L, Sullivan EE, Phillips RS (2017). Impact of community health workers on use of healthcare services in the united states: a systematic review. J Gen Intern Med.

[32] Kangovi S, Mitra N, Grande D, Long JA, Asch DA (2020). Evidence-based community health worker program addresses unmet social needs and generates positive return on investment. Health Aff.

[33] Viswanathan M, Kraschnewski JL, Nishikawa B, Morgan LC, Honeycutt AA, Thieda P, Lohr KN, Jonas DE (2010). Outcomes and costs of community health worker interventions: a systematic review. Med Care.

[34] Crespo R, Christiansen M, Tieman K, Wittberg R (2020). An emerging model for community health worker-based chronic care management for patients with high health care costs in rural Appalachia. Prev Chronic Dis.

[35] Peretz PJ, Matiz LA, Findley S, Lizardo M, Evans D, McCord M (2012). Community health workers as drivers of a successful community-based disease management initiative. Am J Public Health.

[36] Shah M, Kaselitz E, Heisler M (2013). The role of community health workers in diabetes: update on current literature. Curr Diab Rep.

[37] Ruis AR, Golden RN (2008). The schism between medical and public health education: a historical perspective. Acad Med.

[38] Westgate C, Musoke D, Crigler L, Perry HB (2021). Community health workers at the dawn of a new era: 7. Recent advances in supervision. Health Res Policy Sys.

[39] Perry HB (2020). Health for the people: National Community Health Programs from Afghanistan to Zimbabwe.

[40] Assegaai T, Schneider H (2019). The supervisory relationships of community health workers in primary health care: social network analysis of ward-based outreach teams in Ngaka Modiri Molema District, South Africa. BMJ Glob Health.

[41] Agarwal S, Perry HB, Long L, Labrique AB (2015). Evidence on feasibility and effective use of mHealth strategies by frontline health workers in developing countries: systematic review. Trop Med Int Health.

[42] Odendaal W, Goudge J, Griffiths F, Tomlinson M, Leon N, Daniels K (2015). Healthcare workers' perceptions and experiences on using mHealth technologies to deliver primary healthcare services: a qualitative evidence synthesis. Cochrane Database Syst Rev.

[43] Källander K, Tibenderana JK, Akpogheneta OJ, Strachan DL, Hill Z, ten Asbroek AH, Conteh L, Kirkwood BR, Meek SR (2013). Mobile health (mHealth) approaches and lessons for increased performance and retention of community health workers in low- and middle-income countries: a review. J Med Internet Res.

[44] Agarwal S, Rosenblum L, Goldschmidt T, Carras M, Goal N, Labrique A (2016). Mobile technology in support of frontline health workers. John Hopkins Univ Glob mHealth Initiat.

[45] Svoronos T, Mjungu P, Dhadialla R (2010). CommCare: Automated quality improvement to strengthen community-based health. Proceedings of the SHOPS mHealth eConference.

[46] Mishra SR, Neupane D, Preen D, Kallestrup P, Perry HB (2015). Mitigation of non-communicable diseases in developing countries with community health workers. Global Health.

[47] White A, Thomas DS, Ezeanochie N, Bull S (2016). Health worker mHealth utilization: a systematic review. Comput Inform Nurs.

[48] Braun R, Catalani C, Wimbush J, Israelski D (2013). Community health workers and mobile technology: a systematic review of the literature. PLoS ONE.

[49] Lygidakis C, McLoughlin C, Patel K (2016). Achieving universal health coverage: Technology for innovative primary health care education. World Organization of Family Doctors (WONCA), iheed.

[50] Bertman V, Petracca F, Makunike-Chikwinya B, Jonga A, Dupwa B, Jenami N, Nartker A, Wall L, Reason L, Kundhlande P, Downer A (2019). Health worker text messaging for blended learning, peer support, and mentoring in pediatric and adolescent HIV/AIDS care: a case study in Zimbabwe. Hum Resour Health.

[51] Pimmer C, Mhango S, Mzumara A, Mbvundula F (2017). Mobile instant messaging for rural community health workers: a case from Malawi. Global Health Action.

[52] Henry JV, Winters N, Lakati A, Oliver M, Geniets A, Mbae SM, Wanjiru H (2016). Enhancing the supervision of community health workers with WhatsApp mobile messaging: qualitative findings from 2 low-resource settings in Kenya. Glob Health Sci Pract.

[53] Barnett S, Jones SC, Caton T, Iverson D, Bennett S, Robinson L (2014). Implementing a virtual community of practice for family physician training: a mixed-methods case study. J Med Internet Res.

[54] Li L, Lin C, Pham LQ, Nguyen DB, Le TA (2022). Networking community health workers for service integration: role of social media. AIDS Care.

[55] Agarwal S, Perry HB, Long L, Labrique AB (2015). Evidence on feasibility and effective use of mHealth strategies by frontline health workers in developing countries: systematic review. Trop Med Int Health.

[56] Braun R, Catalani C, Wimbush J, Israelski D (2013). Community health workers and mobile technology: a systematic review of the literature. PLoS One.

[57] Rahmani A, Lai J, Jafarlou S, Yunusova A, Rivera A, Labbaf S, Hu S, Anzanpour A, Dutt N, Jain R, Borelli J (2020). Personal mental health navigator: harnessing the power of data, personal models, and health cybernetics to promote psychological well-being. arXiv.

[58] Hood L, Balling R, Auffray C (2012). Revolutionizing medicine in the 21st century through systems approaches. Biotechnol J.

[59] (2021). MOMS Orange County.

[60] Guo Y, Lee J, Rousseau J, Pimentel P, Bojorquez Y, Cabasag C, Silva M, Olshansky E (2016). Potential economic impact of a coordinated home visitation program. Calif J Health Promot.

[61] Guo Y, Pimentel P, Lessard J, Rousseau J, Lee J, Bojorquez Y, Silva M, Olshansky E (2016). A community-based home visitation program's impact on birth outcomes. MCN Am J Matern Child Nurs.

[62] (2021). Welcome to the UNITE Project - Two happy hearts. UNITE.

[63] Jimah T, Borg H, Mehrabadi MA, Labbaf S, Guo Y (2021). A digital health approach to promote emotional well-being in pregnant women: the two happy hearts case study. Proceedings of the Association of Women's Health, Obstetric and Neonatal Nurses (AWHONN) Conference.

[64] Okolo C, Kamath S, Dell N, Vashistha A (2021). “It cannot do all of my work”: community health worker perceptions of AI-enabled mobile health applications in rural India. Proceedings of the 2021 CHI Conference on Human Factors in Computing Systems.

[65] Hamideh D, Nebeker C (2020). The digital health landscape in addiction and substance use research: will digital health exacerbate or mitigate health inequities in vulnerable populations?. Curr Addict Rep.

